# Future burden of non-communicable diseases attributable to overweight in Chile: a multistate life table modeling study

**DOI:** 10.1186/s12889-023-16255-w

**Published:** 2023-07-12

**Authors:** Rodrigo Fuentes, Eduardo Nilson, Leandro F. M. Rezende, Diego Giulliano Destro Christofaro, Danilo R. Silva, Paloma Ferrero-Hernández, Carlos Cristi-Montero, Adilson Marques, Claudio Farías-Valenzuela, Gerson Ferrari

**Affiliations:** 1 Universidad de Santiago de Chile (USACH), Escuela de Ciencias de la Actividad Física, el Deporte y la Salud, Santiago, Chile; 2 Center for Epidemiological Research in Nutrition and Public Health, University of São Paulo, São Paulo, Brazil; 3 Oswaldo Cruz Foundation (Fiocruz/Brasilia), Brasília, Brazil; 4 Department of Preventive Medicine, Escola Paulista de Medicina, Universidade Federal de São Paulo, Sao Paulo, Brazil; 5 Physical Education Department, Graduate Program in Movement Sciences, School of Technology and Sciences, São Paulo State University (Unesp), São Paulo, Brazil; 6 Department of Physical Education, Universidade Federal de Sergipe - UFS, São Cristóvão, Brazil; 7 Escuela de Pedagogía en Educación Física, Facultad de Educación, Universidad Autónoma de Chile, Santiago, 8900000 Chile; 8 IRyS Group, Physical Education School, Pontificia Universidad Católica de Valparaíso, Valparaíso, Chile; 9 CIPER, Faculdade de Motricidade Humana, Universidade de Lisboa, Cruz Quebrada, 1499-002 Portugal; 10 Facultad de Ciencias Para El Cuidado de La Salud, Universidad San Sebastián, Lota 2465, Providencia, 7510157 Chile

**Keywords:** High body mass index, Overweight, Obesity, Mortality, Non-communicable diseases, Modeling study

## Abstract

**Background:**

Previous studies have quantified the current burden of diseases attributable to overweight in Chile. However, no study has estimated the attributable burden of overweight in the future. Herein, we estimated the potential impact of different trajectories in the prevalence of overweight on the incidence and mortality from non-communicable diseases (NCDs) in Chilean adults from 2019 to 2030.

**Methods:**

A multistate life table modelling was used to estimate the business-as-usual (BAU: if the current rate of increase in BMI persist through the next 11 years; i.e., 0.4% per year from 2003 to 2017) and three counterfactual scenarios (1: the increase rate of overweight is reduced by half; 2: maintanance of the current prevalence of overweight; 3: the prevalence of overweight is reduced by 6.7%) over a 11-year simulation period for burden of NCDs attributable to overweight in Chilean adults aged 20 to 80 years. The model inputs included nationally representative data of body mass index, national official demographic records, NCDs from the Global Burden of Disease study in 2019, and relative risks from a published meta-analysis.

**Results:**

If the current trends of increase in overweight are maintained in Chile, approximately, 669 thousand cases and 117 thousand deaths from NCDs will occur from 2020 to 2030. In case the increase rate of overweight is reduced by half during this period, around 7 thousand cases and 1.4 thousand deaths from NCDs would be prevented, while achieving no increase in the prevalence of overweight would avert 10 thousand cases and 2 thousand deaths. In the optimistic scenario of reducing the prevalence of overweight by 6.7% until 2030, approximately 25 thousand cases and 5 thousand deaths from NCDs would be prevented.

**Conclusion:**

We estimated that the number of NCDs cases and deaths that could be avoided by decreasing the prevalence of overweight in Chilean adults. Preventive programs aimed to reduce overweight may have a high impact on the future burden of NCDs in Chile.

## Background

Non-communicable diseases (NCDs) remain the major causes of death in the world [1, 2]. Poor dietary factors have been associated with a range of NCDs and thus considered a major contributor to NCDs mortality worldwide [3]. The double burden of poor dietary factors is a global challenge, considering that obesity represents both a disease by itself and a risk factor for several NCDs [4].

Overweight is defined as abnormal or excessive fat accumulation that presents a risk to health [5]. A body mass index (BMI) ≥ 25 kg/m^2^ has been used as a threshold to classify overweight, and ≥ 30 kg/m^2^ obesity [5]. In 2016, more than 1.9 billion adults aged 18 years or older (39%) were living with overweight and over 650 million (13%) with obesity [5]. In Chile, the prevalence of overweight in adultswas as high as 75.6% in 2016-2017 [6]. Previous epidemiologic studies suggest that high BMI is responsible for several NCDs deaths in Chile, such as cardiovascular diseases, cancer and respiratory diseases [7, 8]. It has been estimated that 21.977 deaths from NCD were attributable to high BMI in the country, which represented about 31.6% of major NCD deaths and 20.4% of total deaths in 2018 [7]. Both studies [7, 8] estimated the burden of NCDs attributable overweight by considering a comparative risk assessment model [9]. This model allows to quantify the absolute number and proportion of NCD deaths attributable to overweight, which would have been prevented if the risk factor—in this specific case, high BMI—had been maintained at ideal levels (22 kg/m^2^) [10]. However, the quantification of the future burden of NCDs attributable to overweight in Chile is unavailable. The proportional multistate lifetables model is a well-known and widely recognized method for quantifying the projected burden of diseases under different counterfactual scenarios of exposures [11]. This information is timely and important to inform the development of interventions and public health policies aimed at counteracting the burden of disease caused by high BMI.

In this study, we obtained BMI data from National Health Surveys (NHS) [12–14], relative risk (RR) from a published meta-analysis [15] and epidemiological measures of 11 diseases to estimate the potential impact of reducing the prevalence of overweight and BMI mean on the prevention of NCDs (incidence and mortality) in the Chilean adults aged 20 to 80 years from 2019 to 2030.

## Methods

This study was based on the three available NHS of Chile conducted in 2003, 2009–2010, and 2016–2017. The NHS are a large, nationally representative, population-based study of health behaviors conducted every six years in both urban and rural zones [12–14]. Data for each survey were collected by trained staff where participants responded to questionnaires, and measured anthropometrical and physiological parameters, as well as biological samples. A stratified multistage probability sample of participants aged ≥ 15 years was recruited. The sample included non-institutionalized participants (Chilean or foreign), located in urban and rural areas of the fifteen regions of Chile. Through the Kish algorithm, one participant per household was randomly selected. Sampling weights from the survey accounted for differences in selection probability and non-response rates, and the post-stratification adjustment allowed to expand the sample to the estimated inhabitants in Chile. The final analytical sample of this study included Chilean adults from both sexes, aged 20 to 80 years, participants of the NHS. Details of the each survey of NHS have been published elsewhere [12–14].

The protocol of this study was approved by the Ethics and Research Committee of the Universidad de Santiago de Chile (USACH) (records no. 224/2022). The NHS were conducted by the Chilean Ministry of Health and the protocol was approved by the Ethics Committee of the Pontificia Universidad Católica de Chile (Pontifical Catholic University of Chile—(No. 16–019). A statement to confirm that all methods were carried out in accordance with relevant guidelines and regulations was obtained. Participants signed an informed consent to take part in the study.

BMI at baseline were calculated using NHSs data and counterfactual scenarios represented different scenarios on how trends could evolve and influence BMI by 2030. A business-as-usual (BAU) scenario was modeled, in which the current rate of increase in BMI (0.4% per year from 2003 to 2017) is maintained through the next 11 years, as well as three additional counterfactual scenarios in the study: First, as intermediate scenario, in which the increase rate of BMI is reduced by half of that observed from 2003 to 2017 through the next 11 years (0.2% per year); second, an optimistic scenario with the maintenance of the current prevalence of overweight in Chile (on average, 76.9%); and third, a very optimistic scenario, in which the prevalence of overweight among adults is reduced by 6.7% from 2019 to 2030 (0.61% reduction per year), equivalent to that set by the United States of America in the Healthy People 2030 Plan [16].

### Multistate lifetable modelling

This modelling study was conducted using an adaptation of a validated multistate lifetable  model (MSLT) built in Excel to estimate the incidence, prevalence, and mortality of diseases over the lifetime of Chilean adults [11]. Within the model, the progression of the inhabitants is simulated through four health states: healthy, diseased, dead from the disease and dead from other causes, where the progression through the conditions are founded on rates of incidence, remission, case fatality and death. In the model, changes in the occurrence and mortality of diseases affect the overall number of individuals who remain alive among the inhabitants over time.

The data inputs to the MSLT model were age- and sex-specific and included residents and mortality rates for the baseline inhabitants, extracted from the Official Deaths Statistics of the Ministry of Health from Chile [17]. Disease-specific incidence, mortality, case-fatality rates and prevalence of 11 diseases (Table 1) in Chile in 2019 were obtained from the Global Burden of Disease (GBD) study from a combination of national registration statistics and disease-specific studies. Epidemiological parameretes extracted from GBD were adjusted using DISMOD II [18, 19].

**Table 1:** Description of the multistate life-table model input parameters to estimate the burden of non-communicable diseases from 2019 to 2030 attributable to overweight in Chile

**Model inputs**	**Body mass index (RR per 5 units) **[20]	**Source**
*Baseline characteristics*		
Demographics		National Health Survey of Chile [12–14]
Deaths		Official Deaths Statistics of the Ministry of Health [17]
Prevalence of overweight		National Health Surveys of Chile [12–14]
*Cardiovascular diseases*		
Coronary heart disease	35–59 years: 1.50 (1.39,1.62) 60–69 years: 1.40 (1.32, 1.49) 70–79 years: 1.31 (1.23, 1.40) 80–89 years: 1.30 (1.17, 1.45)	[15]
Stroke	35–59 years: 1.76 (1.52, 2.04) 60–69 years: 1.49 (1.34, 1.67) 70–79 years: 1.33 (1.19, 1.48) 80–89 years: 1.10 (0.94, 1.30)	[15]
Hypertensive heart disease	BMI 15–25: 1.17 (0.77, 1.76) BMI 25–50: 2.03 (1.75, 2.36)	[15]
*Type 2 diabetes*	BMI 15–25: 0.96 (0.59, 1.55) BMI 25–50: 2.16 (1.89, 2.46)	[15]
*Chronic kidney disease*	BMI 15–25: 1.14 (0.74, 1.77) BMI 25–50: 1.59 (1.27, 1.99)	[15]
*Cirrhosis*	BMI 15–25: 0.73 (0.54, 1.00) BMI 25–50: 1.79 (1.54, 2.08)	[15]
*Cancers*		
Colorectal	Men: 1.24 (1.20, 1.28) Women: 1.09 (1.05, 1.13)	[15]
Kidney	Men: 1.24 (1.20, 1.28) Women: 1.09 (1.05, 1.13)	[15]
Liver	35–79 years: 1.47 (1.26, 1.71)	[15]
Breast	Women (> 60 years): 1.12 (1.08, 1.16)	[15]
Pancreas	1.10 (1.07, 1.14)	[15]

*RR* Relative risk, *BMI* body mass index

The MSLT model simulated the impact of changes in the prevalence of overweight over the lifetime of the Chilean adult’s residents. The measures of output contained new incident cases and deaths from 11 diseases that would be averted or delayed. The model also estimated all-cause mortality and morbidity rates by sex and age. Running alongside this central life table were 11 BMI-related disease life tables, where amounts of the population simultaneously resided: coronary heart disease, stroke, hypertensive heart disease, type 2 diabetes, chronic kidney disease, cirrhosis, and types of cancers (i.e., colorectal, kidney, liver, breast and pancreas). The proportion of the Chilean adult residents in each disease life table was a function of the disease incidence and case fatality and including remission for the case of cancers.

The change in BMI was then combined with relative risks (RR) for the association between BMI and diseases through the potential impact fractions (PIFs) equation, that alter the inflow to the BMI-related disease life tables. The RR fo each of the 11 BMI-related diseases per 5 kg/m^2^ increase in BMI by sex and age-groups, whenever available, were obtained from a meta-analysis of 57 cohort studies with a total of 900,000 adults [20]. Time lags from change in BMI to disease incidence were integrated assuming 5 years for cardiovascular diseases, type 2 diabetes, chronic kidney disease and cirrhosis, and 10 years for cancers (Table 1). Other variables that could potentially confound or modify the effect overweight were assumed to remain unchanged in the analysis.

The uncertainty analysis (Monte Carlo) was integrated in the model to estimate probabilistic 95% uncertainty intervals (95% UI) for all model outputs, based on 1,000 draws from specified probabilistic distributions for the model input variables using the Ersatz add-in [21]. This also allows the model to incorporate the usual random error (sampling error) of the RR and exposure prevalence, as well as other potential sources of uncertainty such as residual confounding or portability of the RRs from the meta-analyses to the Chilean population [22].

## Results

Between 2003 and 2016, the average BMI increased 4.2% in men (26.9 kg/m^2^ (95% CI: 21.5, 32.3) in 2003 to 28.1 kg/m^2^ (95% CI: 21.5, 33.5) in 2016) and 4.4% in women (28.1 kg/m^2^ (95% CI: 22.6, 33.5) in 2003 to 29.4 kg/m^2^ (95% CI: 24.0, 34.8) in 2016). The prevalence of overweight increased from 67.8% in 2003 to 75.7% in 2016, with a higher increase from 2009–2016 (vs. previous years) and in women (vs men).

In Chile, if these current trends of BMI increase are maintained from 2019 to 2030, approximately, 10,090 (95% CI: 8,650 to 11,813) new NCD cases are estimated to occur attributable to overweight over this period. The burden on the incidence cases of disease would be higher for diabetes and chronic kidney disease, contributing to 38.2% and 31.9% of the overall overweight-attributable cases, respectively (Table 2).

**Table 2:** Cases (95%CI) of non-communicable diseases attributable to changes in the prevalence of overweight in the business-as-usual (BAU) and in three counterfactual scenarios over a 11-year simulation period (2019 to 2030) for Chilean adults aged 20 to 80 years

Non-communicable diseases	**BAU**	**Scenario 1**	**Scenario 2**	**Scenario 3**
*Cardiovascular diseases*				
Coronary heart disease	53,463 (49,543 to 57,741)	52,729 (48,862 to 56,947)	52,432 (48,587 to 56,626)	50,877 (47,146 to 54,947)
Stroke	79,859 (68,969 to 92,564)	78,889 (68,131 to 91,439)	78,.493 (67,789 to 90,981)	76,499 (66,067 to 88,670)
Hypertensive heart disease	9,091 (7,837 to 10,569)	9,008 (7,766 to 10,473)	8,974 (7,736 to 10,433)	8,796 (7,583 to 10,226)
*Type 2 diabetes*	302,103 (264,342 to 344,064)	299,371 (261,952 to 340,953)	298,243 (260,965 to 339,669)	292,819 (256,219 to 333,491)
*Chronic kidney disease*	199,638 (159,46 to 249,870)	197,349 (157,636 to 247,005)	196,416 (158,910 to 245,837)	191,547 (153,002 to 239,743)
*Cirrhosis*	1,.630 (12,587 to 17,001)	14,439 (12,422 to 16,778)	14,359 (12,350 to 16,685)	14,046 (12,085 to 16,322)
*Cancers*				
Colorectal	4,819 (4,657 to 4,980)	4,757 (4,597 to 4,916)	4,709 (4,551 to 4,866)	4,444 (4,295 to 4,593)
Kidney	1,060 (985 to 1,140)	1,047 (974 to 1,126)	1,038 (965 to 1,116)	984 (915 to 1,058)
Liver	1,466 (1,256 to 1,705)	1,450 (1,243 to 1,687)	1,438 (1,233 to 1,673)	1,370 (1,174 to 1,593)
Breast	1,860 (1,790 to 1,926)	1,839 (1,773 to 1,905)	1,822 (1,757 to 1,887)	1,731 (1,669 to 1,793)
Pancreas	1,111 (1,081 to 1,152)	1,097 (1,067 to 1,137)	1,086 (1,057 to 1,126)	1,022 (994 to 1,059)
Total cancer	10,316 (9,770 to 10,903)	10,190 (9,654 to 10,771)	10,093 (9,563 to 10,668)	9,551 (9,047 to 10,096)

A business-as-usual (BAU) scenario: the current rate of increase in body mass index is maintained through the next 11 years; Scenario 1: body mass index is reduced to half of that observed from 2003 to 2017 through the next 11 years; Scenario 2: the preservation of the current overweight occurrence in Chile; Scenario 3: the prevalence of overweight in adults is reduced by 6.7% from 2019 to 2030

*CI C*onfidence interval

Comparing the different scenarios (BAU, Scenario 1, Scenario 2, Scenario 3) given the changes in the prevalence of overweight, Scenario 3 would have the greatest impact on the number of incident cases of NCD (644,135 cases; 95% CI: 511,149, 753,495) in comparison to BAU (669,100 cases; 95% CI: 572,515 to 782,712), a reduction of 24,965 NCD cases.

Regarding the attributable deaths, cardiovascular diseases would have the highest impact, with 58.5% of deaths attributable to overweight from 2019 to 2030, followed by diabetes (18.6% of the attributable deaths). We found that if the annual increase rate in the prevalence of overweight was reduced by half (Scenario 1), approximately 1,451 (95% CI: 1.281, 1.647) deaths would be prevented or postponed. Alternatively, if the current goal of maintaining the current prevalence of overweight in adults until 2030 were achieved (Scenario 2), approximately, 2,069 (95% CI: 1,830 to 2,346) deaths would be prevented or postponed. Finally, if the country achieved the optimist goal, that is reducing the prevalence of overweight by 6.7% until 2030 (Scenario 3), approximately 5,073 deaths (95% CI: 3,740 to 4,417) would be prevented or postponed, a reduction of 4.3% of the total deaths attributable to overweight (Table 3).

**Table 3:** Death (95%CI) of non-communicable diseases attributable to changes in the prevalence of overweight in the business-as-usual (BAU) and in three counterfactual scenarios over a 11-year simulation period (2019 to 2030) for Chilean adults aged 20 to 80 years

Non-communicable diseases	**BAU**	**Scenario 1**	**Scenario 2**	**Scenario 3**
*Cardiovascular diseases*				
Coronary heart disease	32,303 (29,934 to 34,887)	31,860 (29,523 to 34,408)	31,681 (29,358 to 34,216)	30,777 (28,520 to 33,239)
Stroke	28,896 (24,956 to 33,493)	28,528 (24,638 to 33,067)	28,379 (24,509 to 32,894)	27,663 (23.,91 to 32,064)
Hypertensive heart disease	7,494 (6,461 to 8,713)	7,424 (6,400 to 8,631)	7,396 (6,376 to 8,598)	7,249 (6,249 to 8,427)
*Type 2 diabetes*	21,796 (19,071 to 24,823)	21,580 (18,883 to 24,577)	21,492 (18,806 to 24,478)	21,082 (18,447 to 24,011)
*Chronic kidney disease*	9606 (7,673 to 12,023)	9,476 (7,569 to 11,860)	9,424 (7,527 to 11,795)	9,174 (7,328 to 11,482)
*Cirrhosis*	10,514 (9,046 to 12,218)	10,373 (8,924 to 12,054)	10,314 (8,873 to 11,985)	10,086 (8,677 to 11,720)
*Cancers*				
Colorectal	3,122 (3,017 to 3,226)	3,081 (2,978 to 3,184)	3,050 (2,948 to 3,152)	2,883 (2,786 to 2,979)
Kidney	805 (748 to 866)	795 (739 to 855)	788 (732 to 847)	748 (695 to 804)
Liver	1,343 (1,151 to 1,562)	1,329 (1,139 to 1,546)	1,318 (1,129 to 1,533)	1,255 (1,076 to 1,460)
Breast	464 (447 to 480)	458 (442 to 475)	454 (438 to 470)	431 (416 to 447)
Pancreas	1,005 (978 to 1,042)	993 (966 to 1,029)	983 (956 to 1,019)	927 (801 to 960)
Total cancer	6,739 (6,341 to 7,176)	6,656 (6,264 to 7,089)	6,593 (6,203 to 7,021)	6,244 (5,953 to 6,650)

A business-as-usual (BAU) scenario: the current rate of increase in body mass index is maintained through the next 11 years; Scenario 1: body mass index is reduced to half of that observed from 2003 to 2017 through the next 11 years; Scenario 2: the preservation of the current overweight occurrence in Chile; Scenario 3: the overweight prevalence among adults is reduced by 6.7% from 2019 to 2030

*CI* confidence interval

**Fig. 1:**
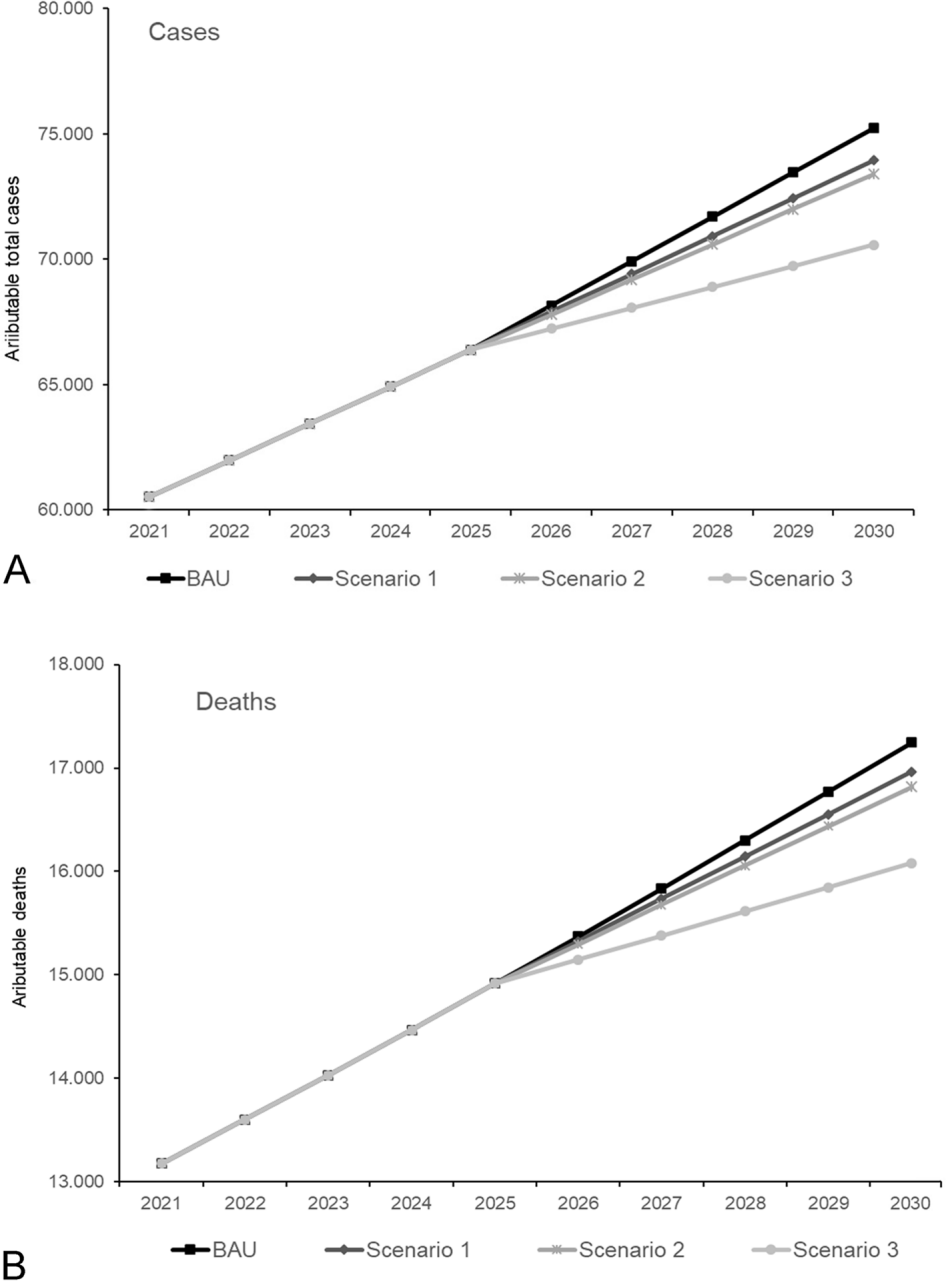
Trends in total NCD cases (**A**) and deaths (**B**) attributable to overweight from 2019 to 2030, considering the continuity of the current trajectory of BMI increase (BAU–business as usual) and different counterfactual scenarios of changes in the prevalence of overweight in Chile

The trends in the total NCD cases and deaths attributable to overweight in Chile during 2019 to 2030 are showed in Fig. 1. The attributable NCD cases will steadily increase in all scenarios until the year 2025, when the lag-times for cardiovascular diseases, diabetes, chronic kidney disease and cirrhosis are completed in the model and the different impact of the changes in BMI for each scenario are evidenced. The Scenario 1 represented the smallest change in the burden of overweight compared to the BAU, while halting the increase in the prevalence of overweight (Scenario 2) would represent a higher impact on NCD cases and deaths. Reducing the prevalence of overweight by 6.7% (Scenario 3) would represent the most important reduction of the burden of overweight-related disease.

## Discussion

In this study, we quantified the potential impact of differences trajectories in the prevalence of overweight on the incidence and mortality from NCDs in the Chilean resident’s population from 2019 to 2030. We estimated that if BMI continues to increase in the next decades as observed in previous years (BAU), the total burden of NCDs cases attributable to BMI will be as high as 669,100 cases between 2020–2030, with the highest number of cases of diabetes, chronic kidney disease and cardiovascular disease. Regarding the number of deaths from NCDs, we estimated that 117,348 deaths may occur during the same period under the BAU scenario of BMI. We estimated that up to 24,965 NCD cases and 5,073 could be avoided in the same period by reducing the prevalence of overweight by 6.7%.

Similarly, a previous study conducted by our group have quantified the future (2020–2030) burden of NCDs attributable to BMI in Brazil. The results showed the potential, impact of different scenarios of decrease in annual increase rate of overweight on the burden of disease, suggesting that policies and interventions aiming at reducing BMI are imperative for the prevention of NCDs in the country [11].

The health effects of overweight are well-described in the literature [4]; therefore, it becomes necessary to estimate the overweight-related disease burden in Chile, as other countries have done [23, 24]. In general, the results suggest that if the current increase in BMI, according to the BAU model is maintained by 2030, Chile will have a burden of diseases attributable to overweight of around 2,602,544 new cases, which, added to the existing cases [25], will represent a heavy burden on the health economic system. Previous studies among Chinese adults have also estimated the future burden of overweight, projecting that the prevalence of overweight by 2030 would be 70.5% and the number of adults having NCDs would be over 810 million [26].

From the eleven diseases considered in our study, diabetes, kidney disease, and cardiovascular diseases represented the highest number of attributable-cases, followed to a lesser extent by cirrhosis and some types of cancer such as breast, colorectal, pancreatic, liver, and kidney. In Chile, cardiovascular diseases have historically been associated with high mortality; even so, it is necessary to highlight the increase of different types of cancer, being today the leading cause of death and known as to be influenced by lifestyle risk factors and overweight [8].

When analyzing the different scenarios proposed in this study, given an intermediate scenario (Scenario 1) where the increase rate of BMI would decrease by half of that observed between 2003 and 2017, the trend is similar to the BAU model, with a total of 661,975 new cases that can be estimated and where disease dynamics follows a similar predominance. Apparently, making changes on the growth curve of the obesity rate does not have a significant impact on the decrease in the number of cases. A second, more optimistic scenario (Scenario 2), in which the current prevalence of overweight is maintained during 2019 and 2030, a total of 659,010 new cases would occur. Finally, a third scenario (Scenario 3), very optimistic, would be the one in which the current prevalence of overweight is reduced by 6.7% from 2019 to 2030. Under this scenario, the number of new cases is estimated to be 644,135. Therefore, these results suggest that reducing the prevalence of overweight, rather and maintaining our reducing the increase rate, could be used as a target by means of prevention of NCDs in Chilean population until 2030.

In this study we also estimated the number of deaths from NCD attributable to overweight under different scenarios of prevalence of overweight between 2020 to 2030. We found that under the BAU model, 117,348 deaths from NCDs attributable to overweight may occur in the country during this period. Now cardiovascular diseases, that is coronary heart disease and stroke, showed the highest number of attributable-deaths. Type 2 diabetes represented the second cause of attributable death, followed by cirrhosis, chronic kidney disease, hypertension and to a lesser extent to different types of cancer. When analyzing Scenario 1, where the rate of increase in BMI is halved between 2019 and 2030, we estimated that a total of 115,897 deaths from NCDs attributable to overweight, representing a slight decrease in the number of deaths under this trend. Assuming Scenario 2, where the current prevalence of overweight is maintained, the number of attributable deaths was estimated to be 115,279, which represented a greater decrease than the BAU model and Scenario 1. Finally, Scenario 3, where it is proposed to reduce the prevalence of overweight by 6.7%, a total of 112,275 deaths from NCDs attributable to overweight would occur. These novel findings suggested that although a more effective intervention will be the one that sets a more ambitious goal, such as Scenario 3, there are differences in morbidity and mortality in the different types of diseases. The most significant impact on morbidity of overweight seems to be due to type 2 diabetes, which prevalence in Chile reaches 12.3% [12]. This would have a great impact on Chilean burden considering its high treatment costs [27] and increasing prevalence in Chile [5]. In this sense, it seems important to consider the consequence of overweight as an objective within the programs that seek to control and improve the levels of obesity in Chile. This is also the case of chronic kidney failure, whose prevalence in Chile is low at around 3.5% of the adult population [12], however it is associated with high treatment management costs [28]. Moreover, overweight have a great impact on mortality associated with cardiovascular diseases, which have long been the main cause of death in Chile and the world [1, 2, 17].

In this nation, a set of strategies and public policies have been implemented that seek to reduce overweight. From a nutritional perspective, in 2016 Law 20606 was promulgated, which incorporates a nutritional labeling system through "warning stamps", considered graphic representations that explicitly declare the amount of sugar, saturated fatty acids and sodium content in foods. This regulation forced many companies to modify the nutritional content of their foods so that they do not have warning labels [29]. In order to improve the nutritional status and physical condition in the Chilean population, in 2004 the "Healthy life" program (Vida sana, in Spanish) was created, which from 2015 acquired the name of "Choose Healthy life" (Elige Vida sana, in Spanish) [30], local initiative made up of multidisciplinary teams of professionals, who have shown effective results in reducing body weight (> 5%) in those people who achieved adherence to the intervention [31].

## Conclusions

In conclusion, we estimated that 669 thousand new cases of NCDs and 117 thousand deaths attributable to overweight may occur between 2019 and 2030 in Chile, if the prevalence of overweight continue to increase as observed in the previous decades. Reducing the prevalence of overweight by 6.7% would decrease, approximately, 25 thousand NCD cases and 5 thousand NCD deaths in Chilean adults between 20 and 80 years of age.

Our modelling analyses suggested that weight control may have a strong potential to reduce the burden of NCDs in the country, and thus improving population health. Strong public health policies aimed to reduce obesogenic environments, together with health and nutrition education are needed to decrease overweight and their comorbidities, encouraging healthier diets and food environments.

## Data Availability

Data may be obtained from a third party and are publicly available. This study is based in part on data from Chilean National Health Survey and Chilean Ministry of Health. More information is available on the website: http://epi.minsal.cl/encuesta-ens-descargable/. For further information, please contact the corresponding authors in the first instance.
